# Green synthesis of Pd nanoparticles supported on reduced graphene oxide, using the extract of *Rosa canina* fruit, and their use as recyclable and heterogeneous nanocatalysts for the degradation of dye pollutants in water

**DOI:** 10.1039/c8ra03404d

**Published:** 2018-06-07

**Authors:** Saba Hemmati, Lida Mehrazin, Hedieh Ghorban, Samir Hossein Garakani, Taha Hashemi Mobaraki, Pourya Mohammadi, Hojat Veisi

**Affiliations:** Department of Pharmaceutical Chemistry, Faculty of Pharmaceutical Chemistry, Pharmaceutical Sciences Branch, Islamic Azad University, (IAUPS) Tehran Iran; Department of Chemistry, Payame Noor University Tehran Iran s_organo2007@yahoo.com hojatveisi@yahoo.com

## Abstract

The current study suggests a convenient synthesis of *in situ*, ecofriendly and well-dispersed palladium nanoparticles with narrow and small dimension distributions on a graphene oxide (GO) surface using a *Rosa canina* fruit extract as a stabilizer and reducing agent without the addition of any other stabilizers or surfactants. The as-synthesized nanocatalyst (Pd NPs/RGO) was assessed with XRD, UV-vis, FE-SEM, EDS, TEM, ICP and WDX. The obtained heterogeneous nanocatalyst showed catalytic performance for reducing 4-nitrophenol (4-NP), rhodamine B (RhB) and methylene blue (MB) at ambient temperature in an ecofriendly medium. The catalyst was retained by centrifugation and reused several times with no considerable change in its catalytic performance.

## Abstract 2

We suggests a convenient synthesis of *in situ*, ecofriendly and well-dispersed Pd nanoparticles with narrow and small dimension distributions on graphene oxide (GO) using *Rosa canina* extract as a reducing agent and as the only stabilizer.

## Graphical abstract

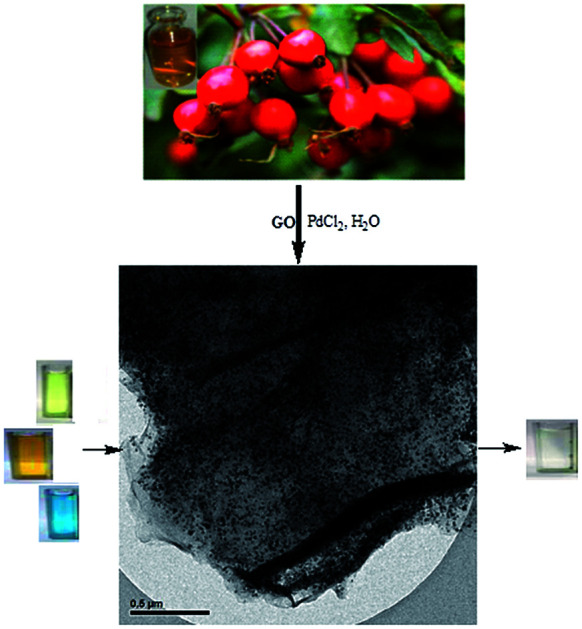

## 1. Introduction

Azo dyes and nitrophenol materials are widely used by different industries including ceramics, cosmetics, textiles, explosives and paper factories. These compounds as carcinogenic, toxic and bio-refractory pollutants are common in wastewaters.^1,2^ The common methods of wastewater treatment which include reverse osmosis, chemical coagulation or adsorption, due to the high resistance, stability and low solubility in water of these compounds, would not be sufficient and effective for their degradation to non-hazardous products.^1–4^ Thus, it would be essential to eliminate these contaminants using an alternative technique. The procedure of chemical reduction in the presence of metal nanoparticles (m-NPs) and NaBH_4_ is among the most presently used approaches for decolorizing or detoxifying these compounds for the elimination of pollutants from wastewater.^5–7^

It has been observed that new m-NPs (such as Pt, Ag, Pd and Au) have particular chemical, physical, thermodynamic and optical features that cause them to be useful in different fields like catalysis,^8*a*–*e*^ biological probing,^10^ diagnostics,^9^ targeted drug delivery,^12^ and sensing.^11^ Moreover, noble m-NPs with high ratios of surface to volume can significantly increase the interactions between catalysts and reactants. In addition, due to their higher catalytic surface area, heterogeneous catalysts are extensively used in the nanoparticle form. Moreover, without difficulty, nanocatalysts can be removed from the products in a reaction mixture, and this creates recyclable catalysts.^8*c*–*e*^

Among diverse m-NPs, Pd NPs have recently attracted much attention due to their abilities to be used as homogeneous or heterogeneous catalysts in different reactions due to their large surface to volume ratio.^8*a*–*e*,13^ However, the agglomeration of m-NPs is unavoidable. An ideal support would be necessary to avoid this m-NPs agglomeration and would aid with the recovery, separation, and stability problems of m-NPs. Various inorganic compounds like Ag,^15^ graphene oxide,^14^ SiO_2_,^17^ TiO_2_,^16^ Fe_3_O_4_ (ref. 18) have been applied as supports for m-NPs. Among different solid supports, graphene and its derivatives are some of the best supports to grow and anchor m-NPs due to their great thermal stabilities, great specific surface areas, appropriate mechanical strengths, light weight properties, good conductivities and excellent adsorption capacities.^19–21^

There are various methods for the synthesis of m-NPs on graphene, reduced graphene oxide (RGO) and graphene oxide (GO) *via* chemical reduction (such as with sodium borohydride, hydrazine hydrate, salicylic acid, oxalic acid, ethylene glycol, NaOH and ascorbic acid) physical techniques, or metal precursors, *etc.*^22^ However, most of them need the application of hazardous chemicals, large amounts of energy, and are extremely expensive which would be unacceptable in the pharmaceutical, medicine and cosmetics industries. Reducing agents may cause harsh chemical absorption on the surfaces of NPs, improving the issues of toxicity. Thus, it is urgent to design a greener and better technique for the synthesis of m-NPs–graphene (GO or/and RGO) hybrids for the use in organic reactions as active catalysts. The green synthesis of m-NPs using non-toxic solvents like biological extracts, water, and environmentally benign techniques is considered as the most attractive aspect of nanoscience and nanotechnology .^4,8*a*–*d*,16,21,23,24^ Furthermore, these methods have simple methodologies, easy work up and high yields, are cost-effective and environment friendly and have organic solvents removal advantages. Among the biological approaches, plant extract mediated biological procedures are useful and simple methods, with no use of costly, harsh and harmful chemicals, for the synthesis of different m-NPs that have been widely used for determining the presence of and removing ionic and dyes pollutants from aqueous media.^8*a*,16,17^ Biological approaches provide an alternative to common physical and chemical methods.^4,8*a*,*c*,*d*,16,21,23,24^

Researchers, up to now, have mainly focused on the application of biowaste materials as supports. The biowaste's low price make it a cheap adsorbent and catalyst support.

**Fig. 1:**
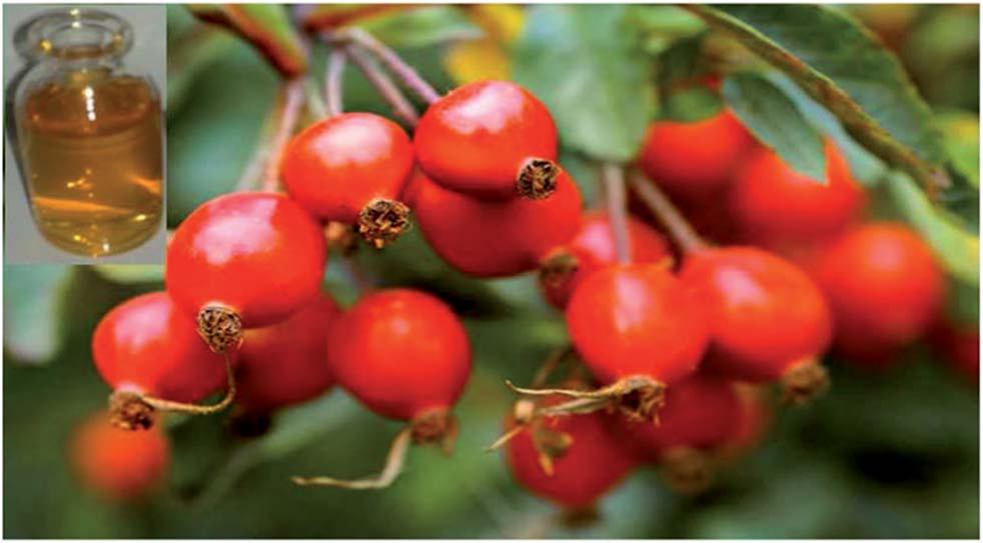
Image of *Rosa canina* fruits.

The genus *Rosa*, with more than 100 types, is extensively spread in the Middle-East, Asia, Europe and America.^8*a*,25^ Mature *Rosa canina* fruit (dog rose) (Fig. 1) has various biologically active compounds, such as organic acids, tocopherol, sugars, amino acids, pectins, fatty acids, flavonoids, tannins, vitamins (particularly vitamin C and other K, B1, B2, PP, D and E vitamins), carotenoids (lycopene and β-carotene), micro- and macro-elements, *etc.* These compounds are found to have antioxidant, anticancer and antimutagenic efficacy and constitute a crucial source of food and medicine for most civilizations. Routine food preparations of rose are jellies, jams, juices, wines and teas, as well as mixtures with caviar.^8*a*,26,27^ The latest research showed that extracts of *Rosa canina* fruit were influential for prevention of biofilm formation and inhibition of growth in methicillin-resistant *Staphylococcus aureus*. The reaction of *Rosa* with biological molecules causes cell and tissue injuries by lipo peroxidation, DNA degradation or proteolysis.^28,29^

Various physical and chemical methods including sonochemistry, electrochemistry, microwave irradiation, solid state reaction, quick precipitation, sol–gel and alcohothermal synthesis have been applied for Pd NP synthesis.^30–32^ However, these methods have some difficulties, including harsh reaction conditions, high temperatures, time-consuming reactions, and the application of costly, poisonous and dangerous stabilizers or capping agents to protect the dimensions and composition of the Pd NPs. The application of organic solvents and the low yields of products are causes of environmental pollution.^4,8*c*,33-35^ Among these methods, the green synthesis of NPs has benefits compared to other methods because of its easy reaction operation, being ecofriendly and cheap, having very mild reaction conditions, being suitable for large-scale fabrication, and the lack of a need to apply high temperatures or pressures, or use organic ligands, surfactants or costly and poisonous materials in the synthetic protocols.^8*a*,23,24,36^

Following our previously studies,^37^ here we report the synthesis of Pd NPs supported on reduced graphene oxide (Pd NPs/RGO) *via* the reduction of Pd^2+^ and GO ions by applying extracts of *Rosa canina* fruit as a stabilizing and reducing agent. The applied strategy provided well-dispersed Pd NPs on the graphene sheets. In addition, the main objectives of this study were to enhance the catalytic performance of the nanocatalyst and to recycle and reuse the nanocatalyst, with or without minimal degradation. The nanocatalyst was studied under mild operating conditions, for the degradation of organic dyes in water in the presence of NaBH_4_ at room temperature, by UV-vis spectroscopy.

## 2. Experimental

### 2.1. *Rosa canina* fruit extract preparation

The fresh *Rosa canina* fruits were thoroughly washed with double-distilled water three times before application. To 100 mL of deionized water, 10 g of fruit was added and for 15 min was boiled in a water bath. The mixture was then filtered and cooled down through Whatman filter paper no. 1 to achieve an aqueous extract. The filtered extract was stored in a refrigerator at 4 °C until required for further application.

### 2.2. The Pd NPs/RGO green synthesis using *Rosa canina* extract

Finally, using the aqueous *Rosa canina* fruit extract, the Pd NPs/RGO was prepared. For the Pd NPs/RGO synthesis, the extract was added into GO, that was obtained from natural graphite powder, using a modified Hummers technique.^38^ Then, 50 mL of 0.3 M PdCl_2_ was added dropwise into the mixture at 80 °C under intensive stirring for 12 h. By centrifugation, the achieved Pd NPs/RGO was separated from the reaction medium, and was washed with deionized water several times, and was then applied in the investigation and characterization of catalysis. The palladium concentration in Pd NPs/RGO was 12.6 wt%, determined by ICP-AES.

### 2.3. The general procedure for 4-NP reduction

As an example, 10 mL of a solution of 2 mM 4-nitrophenol was mixed with 2.0 mg of the Pd NPs/RGO nanocomposite and the mixture was agitated for 1 min at ambient temperature. Next, 5 mL of a prepared NaBH_4_ solution (0.25 M) was poured to the mixture and agitated at ambient temperature until the yellow solution became colorless. The progress of the reaction was recorded using UV-vis spectroscopy. After completion of reaction, the catalyst was isolated through centrifugation and was reused after washing with water and ethanol.

### 2.4. Reduction of methylene blue (MB) and rhodamine B (RhB) by the Pd NPs/RGO nanocomposite

In general, 2.0 mg of the nanocatalyst was poured into 10 mL of an aqueous solution of MB (or RhB) (3 × 10^−5^ M). Next, 25 mL of aqueous NaBH_4_ (4 × 10^−3^ M) was poured into the mixture and the obtained mixture was agitated at ambient temperature. The development of the reaction conversion was examined through recording time-dependent UV-vis absorption spectra of the mixture with a spectrophotometer. After completion of the reaction, the catalyst was simply isolated from the reaction mixture using centrifugation, and was sequentially washed with ethanol and dried for the next cycle.

## 3. Results and discussion

### 3.1. The results of catalyst characterization

In this research, during one step, Pd NPs/RGO was prepared through the reduction of Pd^2+^ ions and graphene oxide using the extract of the *Rosa canina* fruit as a stabilizing and reducing agent. The application of *Rosa canina* fruit extract, as a valuable and economic option, gave an environmentally benign and interesting synthetic route for Pd NPs without the application of hazardous materials and toxic organic solvents. The application of *Rosa canina* fruit extract as a green technique for Pd NPs synthesis was described in this study.^8*a*^

**Fig. 2:**
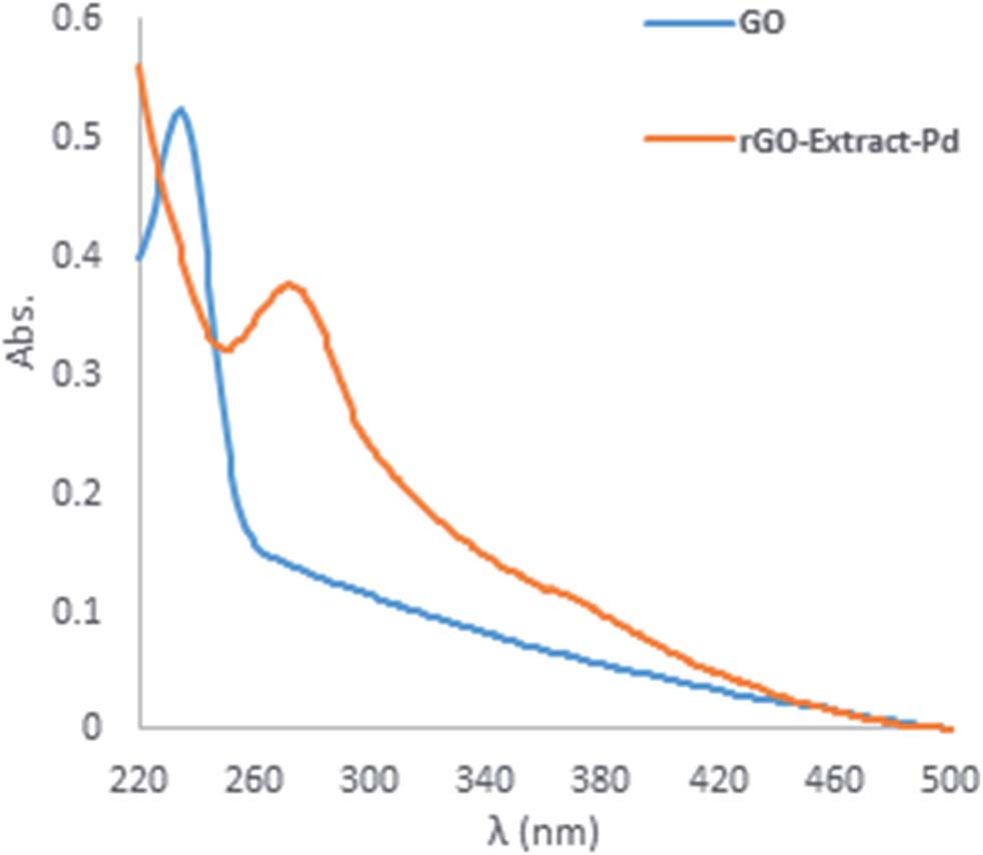
UV-vis spectra of the GO and RGO.

Then, without using toxic, expensive, and harsh chemicals RGO was prepared from GO in the presence of *Rosa canina* fruit extract. The RGO was evaluated using UV-vis spectroscopy. The GO UV spectrum (Fig. 2) indicates a maximum absorption peak (*λ*_*max*_) at 232 nm because of the π → π* transition of the aromatic C–C bonds and a weak shoulder obtained at 300 nm because of the n → π* transitions of the C=O bonds. The green RGO, that was synthesized using the *Rosa canina* fruit, gave a UV-vis spectrum with a new peak and a characteristic peak that red shifted from 232 nm to 270 nm, showing the reaction end and the formation of RGO (Fig. 2).

At last, following this study, we applied the results to prepare Pd NPs/RGO by treating with PdCl_2_ and GO in *Rosa canina* fruit extract for 12 h at 80 °C. The Pd NPs/RGO was evaluated by XRD, TEM, FE-SEM, DLS, WDX and EDS.

**Fig. 3:**
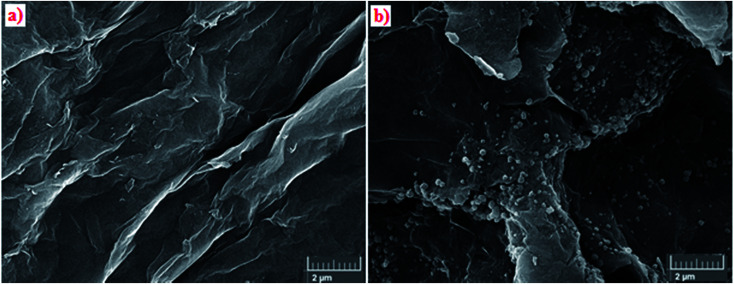
(a) FE-SEM images of GO, and (b) Pd NPs/RGO.

**Fig. 4:**
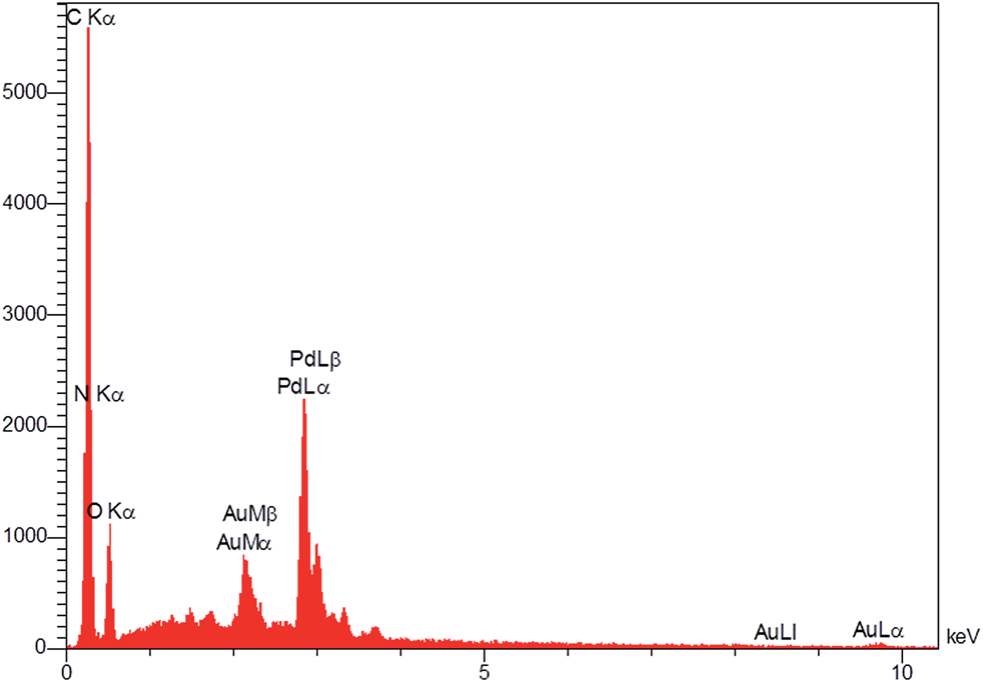
EDX spectrum of Pd NPs/RGO.

Fig. 3a and b show the results of the analysis of the field emission scanning electron microscopy (FE-SEM) performed to evaluate the morphology of GO and Pd NPs/RGO. Fig. 3b is a FE-SEM representative image of the Pd NPs/RGO with large quantities of Pd NP distributed on the surface of the RGO in comparison with the GO. EDX analysis verified the elemental composition of the synthesized nanocomposite (Fig. 4). The strong peak at 3 keV shows the existence of elemental Pd nanoparticles as was clear from previous observations. Apart from Au, the other existing elements, shown by the EDX analysis, were carbon, nitrogen and oxygen.

**Fig. 5:**
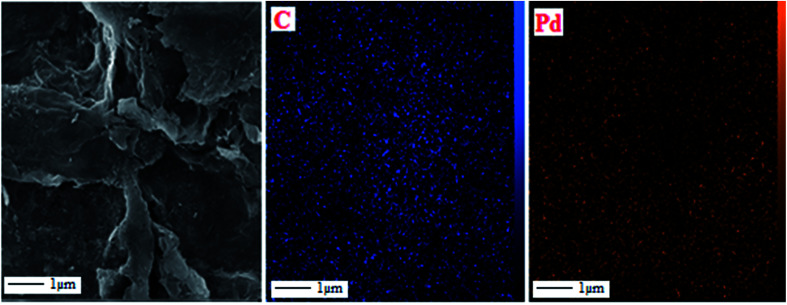
FE-SEM image of Pd NPs/RGO and elemental maps of C, and Pd atoms.

Wavelength-dispersive X-ray spectroscopy (WDX)-coupled with quantified FE-SEM mapping of the sample was studied as well (Fig. 5). The wavelength-dispersive X-ray spectroscopy (WDX) can reveal qualitative data regarding the distribution of various chemical elements in the catalyst matrix. Considering the compositional maps of Pd and C of the Pd NPs, the existence of Pd and C, with suitable dispersion, is evidently characterized in the composite.

**Fig. 6:**
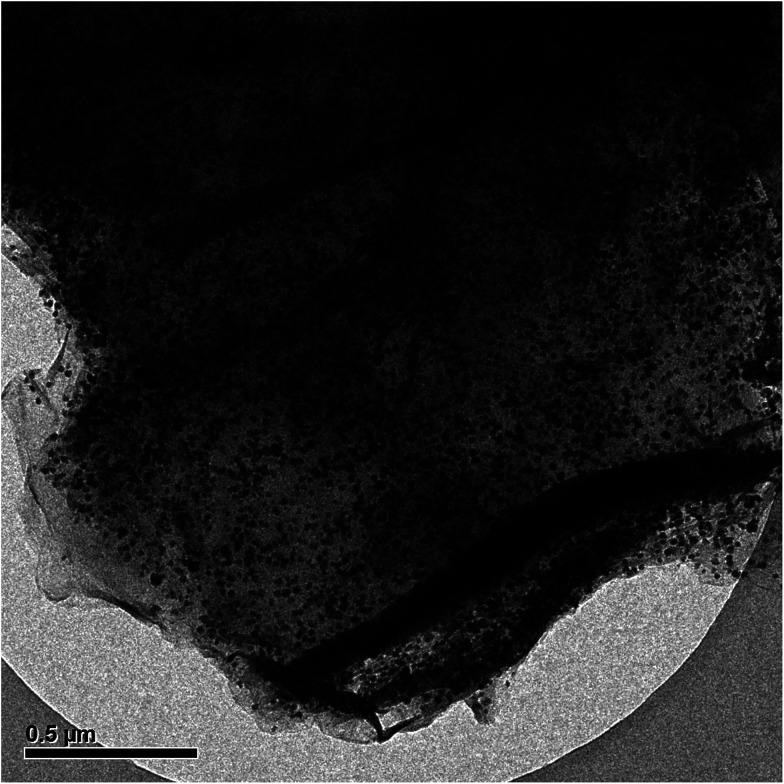
TEM image of Pd NPs/RGO.

Based on TEM analysis (Fig. 6), Pd was well-dispersed on the reduced GO surface. This outcome presented that the *Rosa canina* fruit extract has a main role in increasing the dispersibility of the Pd NPs.

**Fig. 7:**
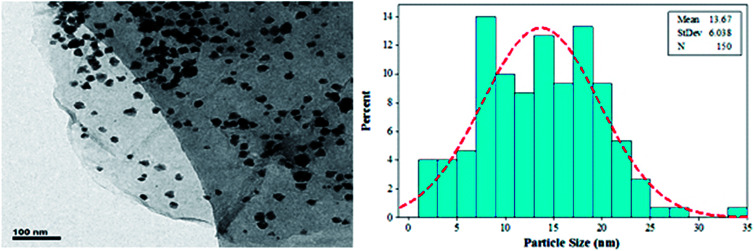
TEM image of Pd NPs/RGO with Pd NP size distributions.

Fig. 7 indicates the Pd NP size distributions. The histogram and TEM images show that the average size of the Pd NPs is approximately 13.67 nm.

**Fig. 8:**
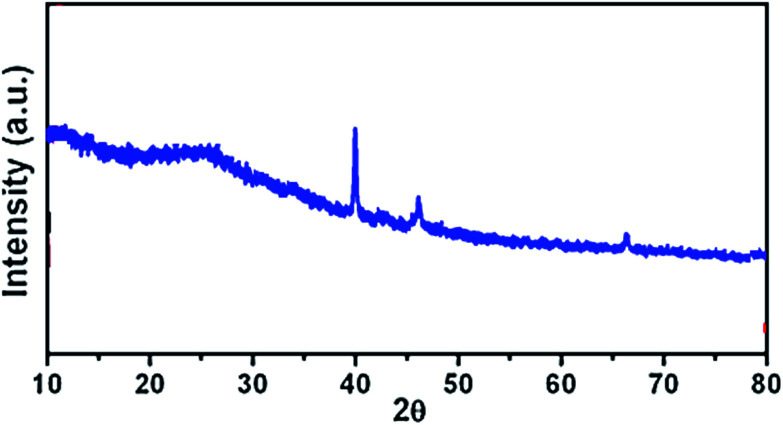
XRD pattern of Pd NPs/RGO.

The XRD pattern of the Pd NPs/RGO can be seen in Fig. 8. XRD analysis indicates a diffraction peak at 2*θ* of 23.4°, (002) relating to the RGO. The XRD pattern indicates major diffraction peaks at 39.7°, 46.5°, 67.3° (2*θ*), which can be indexed to the (111), (200), and (220) planes of face-centered cubic Pd (JCPDS no. 89-4897).

**Fig. 9:**
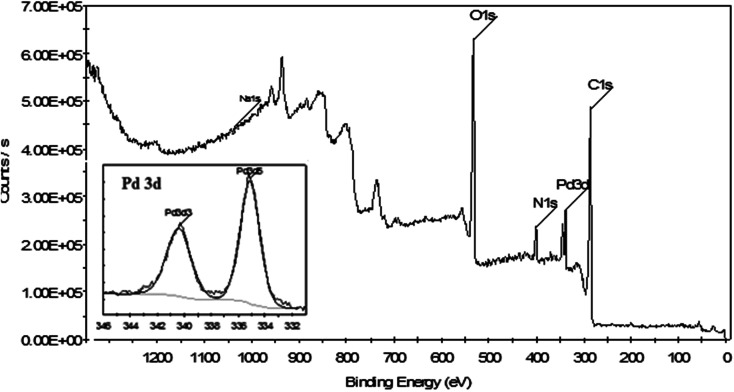
XPS spectrum related to the elemental survey scan of Pd NPs/RGO and in the Pd 3d region (inset).

XPS is a powerful technique for investigation of the electronic features of a species formed on a surface, such as the electron environment, the oxidation state, and the binding energies of the core electrons of a metal. Fig. 9 shows the XPS elemental survey scans of the surface of the Pd NPs/RGO catalyst. The peaks corresponding to oxygen, carbon, nitrogen, and palladium are clearly seen in this spectrum. Moreover, XPS analysis was carried out to determine the oxidation state of the Pd. In Fig. 9, the Pd binding energy of Pd NPs/RGO indicates two peaks centered at 335.26 and 341.64 eV relating to Pd 3d_3/2_ and Pd 3d_5/2_, respectively. According to earlier research,^39^ these peaks relate to Pd(0) species which are capped by groups of biomolecules from biological extracts. In other words, all Pd atoms are shown in their reduced form, which confirms the effective reduction of Pd(ii) to Pd(0) NPs by the extract (Fig. 9, inset). The presence of C 1s and N 1s in the spectrum that resulted from XPS analysis reconfirmed the existence of the extract on the surface of the graphene.

### 3.2. Application of Pd NPs/RGO for *p*-nitrophenol (4-NP), rhodamine B (RhB), and methylene blue (MB) catalytic reduction

In order to investigate the catalytic performance of Pd NPs/RGO, the reduction of *p*-nitrophenol in the presence of NaBH_4_ was chosen as a model reaction for the reductions of rhodamine B (RhB), methylene blue (MB), and *p*-aminophenol.

#### 3.2.1. 4-NP catalytic reduction

**Fig. 10:**
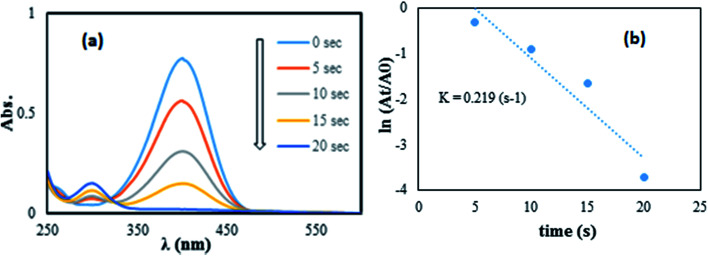
The reduction of 4-NP in aqueous solution recorded every 5 s using the Pd NPs/RGO nanocomposite (1 mg) as a catalyst (a) ln(*A*_*t*_/*A*_0_) *versus* reaction time for the reduction of 4-NP (b).

This reaction is especially simple to detect as there is just one product, *p*-aminophenol (4-AP), and by change in the UV-vis absorbance measured at 300 and 400 nm, the reaction extent can be followed.^30,40^ As shown in Fig. 10a, the absorption peak at 400 nm decreased significantly over 20 s and another peak was observed at 300 nm corresponding to 4-aminophenol. Consequently, it can be stated that 4-NP was completely converted to 4-aminophenol, indicating the reduction of 4-NP to the 4-AP form. The reduction happened from the donor, BH_4_^−^, to the acceptor, 4-NP, after the adsorption of both of them on the surface of the Pd/graphene NPs. A hydrogen atom formed from the hydride attack to reduce the molecules of 4-NP, following the electron transfer to the nanoparticles of Pd.^4^ The degree of reduction, catalyzed by Pd NPs/RGO, was considered to be affected by the NaBH_4_ level, as this reagent was used in great excess compared to 4-NP. Therefore, the kinetic data were fitted using a first-order rate law. Since the absorbance of 4-NP in the environment is proportional to its concentration, the ratio of absorbance at time *t* (*A*_*t*_) to that at the beginning of the reaction (*A*_0_) should be equal to the 4-NP concentration ratio (*C*_*t*_/*C*_0_). A linear relation between reaction time and ln(*C*/*C*_0_) was obtained in the reduction catalyzed with the Pd NPs/RGO nanocomposite (Fig. 10b), and the rate constant of *k* was computed to be 2.19 × 10^−2^ s^−1^.

To illuminate the important benefits and the reliability of the technique of our current research, it was compared with other available methods, and several outcomes for reducing 4-NP are presented in Table 1. The outcomes in Table 1 specify that Pd/graphene is a more effective catalyst with regard to reaction time, when compared with the earlier reported catalysts. Furthermore, the prepared nanocatalyst was reusable, steady, and showed good catalytic performance.

**Table 1:** Comparison of the results of this work with other reported methods in the reduction of 4-NP to 4-AP

Entry	Catalyst	Time	Ref.
1	GA–Pt NPs	8 h	41
2	Resin–Au NPs	20 min	42
3	NAP–Mg–Au(0)	7 min	43
4	Ni–PVAm/SBA-15	85 min	44
5	Ag NPs/seashell	4.5 min	45
6	Ag/TiO_2_ nanocomposite	2 min	16
7	PdCu/graphene	1.5 h	46
8	HMMS–NH_2_–Pd	60 min	47
9	Pd/C	5 min	This work
10	Pd NPs/RGO	20 s	This work

#### 3.2.2. MB and RhB catalytic reduction

**Fig. 11:**
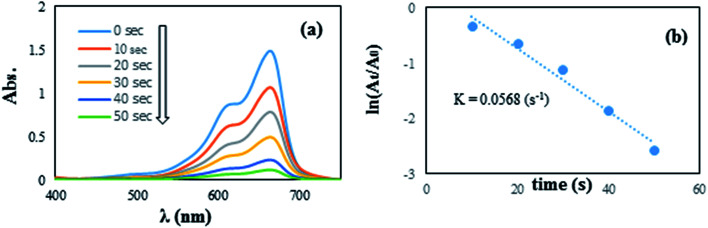
The reduction of MB in aqueous solution recorded every 10 s using the Pd NPs/RGO nanocomposite (1 mg) as a catalyst (a) ln(*A*_*t*_/*A*_0_) *versus* reaction time for the reduction of MB (b).

**Fig. 12:**
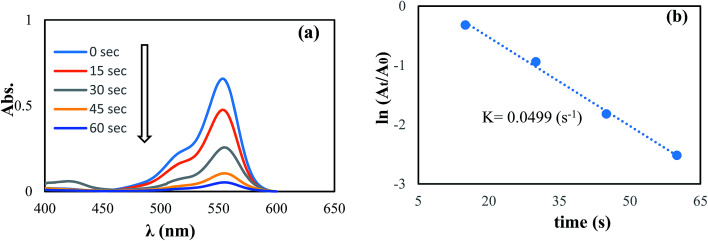
The reduction of RhB in aqueous solution recorded every 15 s using the Pd NPs/RGO nanocomposite (1 mg) as a catalyst (a) ln(*A*_*t*_/*A*_0_) *versus* reaction time for the reduction of RhB (b).

In another step, the catalytic reduction of MB, and RhB dyes with NaBH_4_ was selected to determine the catalyst performance of the synthesized Pd NPs/RGO. By UV-vis spectroscopy, the progress of the reactions were monitored, as shown in Fig. 11 and 12. As can be observed in the UV-vis spectra, in aqueous medium, MB and RhB display peaks at 663 and 554 nm, respectively.

Fig. 11 and 12 show the absorbance observed by time-dependent UV-vis, related to the reduction reactions of the azo dyes. It is obvious that the reduction of the dyes happened immediately, upon adding the Pd NPs/RGO into the diverse dye solutions and the decolorizing was almost finished after 50 and 60 s, for MB and RhB, respectively.

These results suggest that Pd NPs/RGO is the probable cause of the –N=N– double bond cleavage in the chromophoric groups in the structures of the azo dyes and thus decolorizes the solutions.^48^ Moreover, the rate constants for the reduction of RhB and MB were examined. As the NaBH_4_ concentration was much more than that of the azo dyes, it can be considered constant over the course of the reaction. Consequently, based on pseudo first order kinetics, ln(*A*_*t*_/*A*_0_) = *kt*, where *A*_*t*_ represents the dye absorbance at time *t*, *A*_0_ refers to the dye initial absorbance and the slope *k* is the obvious reduction rate constant. Best fitting was carried out for the two rate graphs of the azo dyes, and this indicated linear relations between time and rate (Fig. 11b and 12b). As presented in Fig. 11b and 12b, the computed rate constants for reducing RhB and MB using the graphene/Pd in the presence of NaBH_4_ are 4.99 × 10^−2^ s^−1^ and 5.68 × 10^−2^ s^−1^, respectively.

In our current research study, the outcomes have been compared with a diverse set of reported methods for reduction of different dyes using NaBH_4_ in Table 2 to illuminate the performance and usability of our green catalyst. The reaction times have been presented in Table 2. According to the results, these values are better for our catalyst, compared to those of previous studies of the same catalytic reactions using various nanocatalysts.

**Table 2:** Comparison of results for this work with other reported methods in the reduction of MB and RhB

Dye	Catalyst	Time	Ref.
MB	Porous Cu microspheres	8 min	53
MB	SiNWAs–Cu	10 min	54
MB	Au/Fe_3_O_4_@C	10 min	55
MB	Ag NPs/seashell	2.5 min	45
MB	Pd/C	6 min	This work
MB	Pd NPs/RGO	1 min	This work
RhB	Fe_3_O_4_@PANI@Au	18 min	56
RhB	Au-PANI nanocomposite	15 min	57
RhB	SiNWAs–Cu	14 min	54
RhB	Pd/C	5 min	This work
RhB	Pd NPs/RGO	50 s	This work

The excellent catalytic performance of the Pd NPs/RGO nanocomposite could be achieved due to its small-sized and greatly dispersed Pd NPs. The small size of the Pd NPs produces a great potential difference, and this causes a great reduction rate. Furthermore, a smaller particle size can lead to a greater surface-to-volume ratio with further exposed atoms on the surface that can perform as possible catalytic sites.^49–52^

### 3.3. Recyclability of the catalyst

**Fig. 13:**
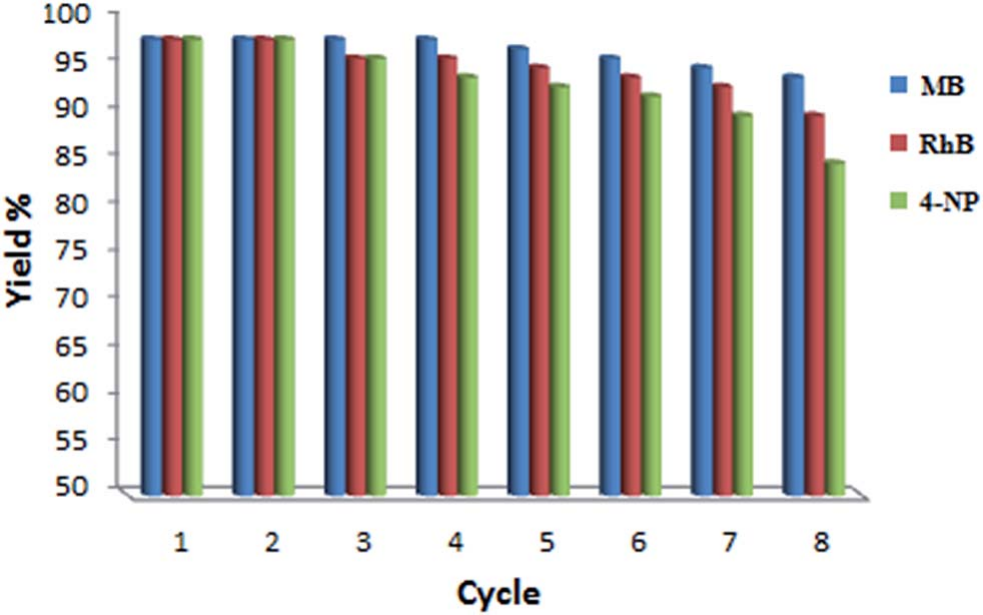
Reusability of the Pd NPs/RGO nanocatalyst for the reduction of MB, RhB and 4-NP in the presence of NaBH_4_.

Although Pd NPs/RGO presented a great catalytic performance, it is vital to investigate the nanocatalyst for its recycle stability. Consequently, Pd NPs/RGO was isolated and reused for degrading the two dyes, RhB and MB, for determination of its effectiveness upon recycling. As shown in Fig. 13, the catalytic performance of Pd NPs/RGO was not reduced considerably after 7 cycles for the degradation of both dyes or 4-nitrophenol, and that indicates the great stability of Pd NPs/RGO. The conversion percentage of both MB and RhB decreased slightly after the catalyst was recycled seven times.

## 4. Conclusions

A facile approach was developed for the synthesis of Pd NPs/RGO using an extract of the *Rosa canina* fruit as a stabilizing and reducing agent. The catalyst was evaluated by UV-vis, FE-SEM, XRD, WDX, ICP, EDX and TEM. Nanoparticles of Pd NPs/RGO showed significant and durable activity for the catalytic reduction of 4-NP, RhB and MB with NaBH_4_ as the hydrogen source in aqueous medium at room temperature. Furthermore, by centrifugation, the catalyst could be simply separated and successively reapplied for seven cycles, without considerable activity loss, with an approximately complete conversion. This technique has the benefits of an easy preparation of the catalyst, high yields, homogeneous catalysts removal, easy methodology and simple work up.

## Conflicts of interest

There are no conflicts to declare.

## Supplementary Material
