# Correction: Green synthesis of Pd nanoparticles supported on reduced graphene oxide, using the extract of *Rosa canina* fruit, and their use as recyclable and heterogeneous nanocatalysts for the degradation of dye pollutants in water

**DOI:** 10.1039/c8ra90060d

**Published:** 2018-07-13

**Authors:** Saba Hemmati, Lida Mehrazin, Hedieh Ghorban, Samira Hossein Garakani, Taha Hashemi Mobaraki, Pourya Mohammadi, Hojat Veisi

**Affiliations:** Department of Chemistry, Payame Noor University Tehran Iran s_organo2007@yahoo.com hojatveisi@yahoo.com; Department of Pharmaceutical Chemistry, Faculty of Pharmaceutical Chemistry, Pharmaceutical Sciences Branch, Islamic Azad University (IAUPS) Tehran Iran

## Abstract

Correction for ‘Correction: Green synthesis of Pd nanoparticles supported on reduced graphene oxide, using the extract of *Rosa canina* fruit, and their use as recyclable and heterogeneous nanocatalysts for the degradation of dye pollutants in water’ by Saba Hemmati *et al.*, *RSC Adv.*, 2018, **8**, 22763–22763.

The affiliations in the original article were transposed; the corrected affiliations are as shown below.

The Royal Society of Chemistry apologises for these errors and any consequent inconvenience to authors and readers.

## Supplementary Material

