# Expression of concern: Green synthesis of Pd nanoparticles supported on reduced graphene oxide, using the extract of *Rosa canina* fruit, and their use as recyclable and heterogeneous nanocatalysts for the degradation of dye pollutants in water

**DOI:** 10.1039/d4ra90110j

**Published:** 2024-09-30

**Authors:** Saba Hemmati, Lida Mehrazin, Hedieh Ghorban, Samira Hossein Garakani, Taha Hashemi Mobaraki, Pourya Mohammadi, Hojat Veisi

**Affiliations:** a Department of Chemistry, Payame Noor University Tehran Iran s_organo2007@yahoo.com hojatveisi@yahoo.com; b Department of Pharmaceutical Chemistry, Faculty of Pharmaceutical Chemistry, Pharmaceutical Sciences Branch, Islamic Azad University (IAUPS) Tehran Iran

## Abstract

Expression of concern for ‘Green synthesis of Pd nanoparticles supported on reduced graphene oxide, using the extract of *Rosa canina* fruit, and their use as recyclable and heterogeneous nanocatalysts for the degradation of dye pollutants in water’ by Saba Hemmati *et al.*, *RSC Adv.*, 2018, **8**, 21020–21028, https://doi.org/10.1039/C8RA03404D.

*RSC Advances* is publishing this expression of concern in order to alert readers that concerns have been raised over the integrity of the data published in this article. The authors have been contacted but have not responded to requests to provide raw data. An expression of concern will continue to be associated with the article until a conclusive outcome is reached.

Signed: Laura Fisher

Date: 16th September 2024

Executive Editor, *RSC Advances*

